# Colitis susceptibility in p47^*phox−/−*^ mice is mediated by the microbiome

**DOI:** 10.1186/s40168-016-0159-0

**Published:** 2016-04-05

**Authors:** E. Liana Falcone, Loreto Abusleme, Muthulekha Swamydas, Michail S. Lionakis, Li Ding, Amy P. Hsu, Adrian M. Zelazny, Niki M. Moutsopoulos, Douglas B. Kuhns, Clay Deming, Mariam Quiñones, Julia A. Segre, Clare E. Bryant, Steven M. Holland

**Affiliations:** Immunopathogenesis Section, Laboratory of Clinical Infectious Diseases, National Institute of Allergy and Infectious Diseases, National Institutes of Health, Bethesda, MD USA; Oral Immunity and Inflammation Unit, Oral and Pharyngeal Cancer Branch, National Institute of Dental and Craniofacial Research, National Institutes of Health, Bethesda, MD USA; Fungal Pathogenesis Unit, Laboratory of Clinical Infectious Diseases, National Institute of Allergy and Infectious Diseases, National Institutes of Health, Bethesda, MD USA; Microbiology Service, Department of Laboratory Medicine, Clinical Center, National Institutes of Health, Bethesda, MD USA; Neutrophil Monitoring Laboratory, Applied/Developmental Research Directorate, Frederick National Laboratory for Cancer Research, Leidos Biomedical Research, Inc., Frederick, MD USA; Translational and Functional Genomics Branch, National Human Genome Research Institute, National Institutes of Health, Bethesda, MD USA; Bioinformatics and Computational Bioscience Branch, National Institute of Allergy and Infectious Diseases, National Institutes of Health, Bethesda, MD USA; Department of Veterinary Medicine, University of Cambridge, Madingley Road, Cambridge, UK

**Keywords:** Chronic granulomatous disease, NADPH, Reactive oxygen species, p47^*phox*^, Microbiome, Inflammatory bowel disease, Colitis, Dextran sodium sulfate

## Abstract

**Background:**

Chronic granulomatous disease (CGD) is caused by defects in nicotinamide adenine dinucleotide phosphate oxidase 2 (NOX2) complex subunits (gp91^*phox*^ (a.k.a. Nox2), p47^*phox*^, p67^*phox*^, p22^*phox*^, p40^*phox*^) leading to reduced phagocyte-derived reactive oxygen species production. Almost half of patients with CGD develop inflammatory bowel disease, and the involvement of the intestinal microbiome in relation to this predisposing immunodeficiency has not been explored.

**Results:**

Although CGD mice do not spontaneously develop colitis, we demonstrate that p47^*phox−/−*^ mice have increased susceptibility to dextran sodium sulfate colitis in association with a distinct colonic transcript and microbiome signature. Neither restoring NOX2 reactive oxygen species production nor normalizing the microbiome using cohoused adult p47^*phox−/−*^ with B6Tac (wild type) mice reversed this phenotype. However, breeding p47^*phox+/−*^ mice and standardizing the microflora between littermate p47^*phox−/−*^ and B6Tac mice from birth significantly reduced dextran sodium sulfate colitis susceptibility in p47^*phox−/−*^ mice. We found similarly decreased colitis susceptibility in littermate p47^*phox−/−*^ and B6Tac mice treated with *Citrobacter rodentium.*

**Conclusions:**

Our findings suggest that the microbiome signature established at birth may play a bigger role than phagocyte-derived reactive oxygen species in mediating colitis susceptibility in CGD mice. These data further support bacteria-related disease in CGD colitis.

**Electronic supplementary material:**

The online version of this article (doi:10.1186/s40168-016-0159-0) contains supplementary material, which is available to authorized users.

## Background

Chronic granulomatous disease (CGD) is a genetic immunodeficiency caused by defects in any one of the five subunits of the nicotinamide adenine dinucleotide phosphate (NADPH) oxidase 2 (NOX2) complex including gp91^*phox*^ (a.k.a Nox2) (*CYBB* (cytochrome b-245, beta polypeptide)), p47^*phox*^ (*NCF1* (neutrophil cytosolic factor 1)), p67^*phox*^ (*NCF2* (neutrophil cytosolic factor 2)), p22^*phox*^ (*CYBA* (cytochrome b-245, alpha polypeptide)), and p40^*phox*^ (*NCF4* (neutrophil cytosolic factor 4)) [1]. This leads to reduced or absent production of reactive oxygen species (ROS) primarily in phagocytes, which manifests clinically as the early onset of recurrent infections, and marked dysregulation of inflammation [2, 3]. In fact, our recent survey of patients with CGD followed at the National Institutes of Health (NIH) suggests that almost 50 % of these patients suffer from inflammatory bowel disease (IBD) (unpublished data).

It has been established that aberrant interactions between the intestinal microbiota and the immune system may fuel intestinal and systemic inflammation (reviewed in [4, 5]). However, it is unclear whether the absence of phagocyte-derived ROS drives intestinal dysbiosis in either mice or humans and if intestinal dysbiosis is a cause or consequence of CGD gastrointestinal (GI) inflammation. Our objective therefore was to define the contribution of the intestinal microbiota in driving IBD penetrance in the context of CGD as a predisposing monogenetic immunodeficiency.

We induced colitis in p47^*phox−/−*^ mice and used bone marrow chimeras, as well as 16S rRNA sequencing in conjunction with microbiome standardization techniques, to examine the contribution of phagocyte-derived ROS in driving intestinal dysbiosis and to determine the role of the microbiome in modulating colitis susceptibility in CGD mice. We found that although phagocyte-derived ROS modulates intestinal microbiomic and transcriptomic signatures, the intestinal microbiota established at birth has a greater impact on colitis susceptibility in p47^*phox−/−*^ mice then the absence or presence of phagocyte-derived ROS.

## Results

### p47^phox−/−^ mice do not spontaneously develop colitis and their neutrophils do not produce ROS

Untreated p47^*phox−/−*^ mice were monitored daily for 16 months. There were no mortalities, and histological examination of colons showed no evidence of subclinical gastrointestinal disease. p47^*phox−/−*^ colons were found to be similar to those of 8-week-old B6Tac wild-type (WT) mice (Additional file 1: Figure S1). We confirmed that neutrophils isolated from p47^*phox−/−*^ mice do not produce ROS by performing dihydrorhodamine (DHR) oxidation assays before and after stimulation with phorbol 12-myristate 13-acetate (PMA) (Additional file 2: Figure S2).

### p47^phox−/−^ mice have increased susceptibility to DSS colitis

**Fig. 1:**
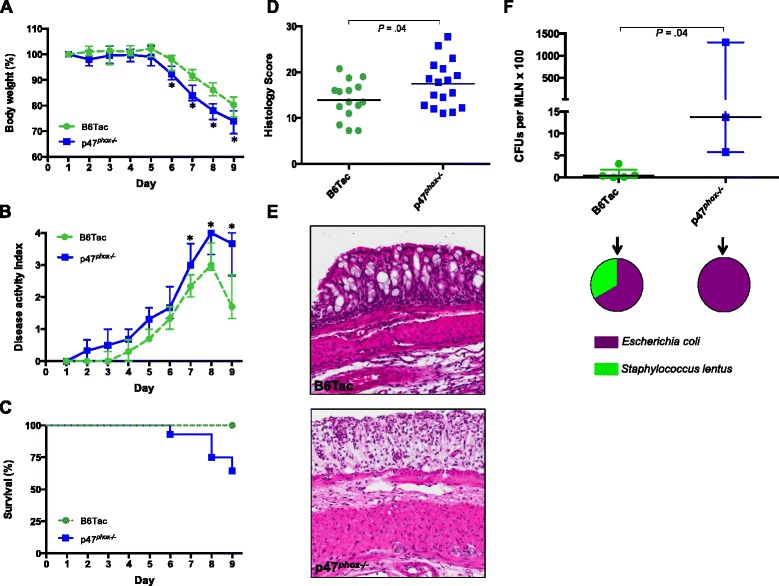
p47^*phox−/−*^ mice have increased susceptibility to DSS colitis. Changes in body weight (**a**), disease activity index (**b**), and survival (**c**) were assessed daily (B6Tac (*n* = 15); p47^*phox−/−*^ (*n* = 28)). Significance was determined using the Mann-Whitney *U* test (*p < 0.05) and log-rank test for survival (*p* = 0.0003). **d** Colonic tissue sections were blindly scored for inflammation, depth of injury, and crypt damage on day 9 (B6Tac (n = 15); p47^*phox−/−*^ (*n* = 17); (*p* = 0.04)). **e** Representative distal colon sections stained with H&E (original magnification X 10). **f** Bacterial translocation to MLNs was determined on day 9. The number of CFUs per MLN is shown (B6Tac (*n* = 5); p47^*phox−/−*^ (*n* = 3); (*p* = 0.04)). *Pie charts* represent the proportion of mice per group where the cultured MLN grew one or more of the listed bacterial species. For all panels, data are representative of 4 independent experiments (except **f**)

We induced acute colitis in p47^*phox*^ mice by administering 3.5 % dextran sulfate sodium (DSS) in drinking water for 7 days followed by 1 day of DSS-free autoclaved water. p47^*phox−/−*^ mice lost more weight than B6Tac and were unable to recover from this weight loss (Fig. 1a). p47^*phox−/−*^ mice had more severe colitis as evidenced by significantly increased disease activity index (DAI) scores after day 6 (Fig. 1b) and 36 % mortality compared to 0 % in B6Tac (*p* = 0.0003) (Fig. 1c). Hematoxylin and eosin (H&E) staining of DSS day 9 colon sections showed severe patchy inflammation characterized by transmural lymphocytic infiltrates, epithelial ulceration, and complete crypt loss, which worsened distally (Fig. 1e). Moreover, histology severity scores were significantly higher in p47^*phox−/−*^ mice (Fig. 1d). To determine whether p47^*phox*^ deficiency contributes to increased epithelial permeability during intestinal inflammation, we assessed bacterial translocation to the mesenteric lymph nodes (MLN) and spleen (data not shown) before and after DSS colitis. p47^*phox−/−*^ mice did not show a spontaneous defect in intestinal permeability, as no bacteria were detected in MLN or spleen prior to treatment with DSS (data not shown). However, p47^*phox−/−*^ mice with DSS colitis had significantly greater numbers of CFUs per MLN (Fig. 1f) and per spleen. To determine whether there was a pattern in the species of translocating bacteria, microbial identification using matrix-assisted laser desorption ionization-time of flight mass spectrometry (MALDI-TOF MS) was performed on all morphologically distinct colonies associated with each MLN. The MLN isolated from both p47^*phox−/−*^ and B6Tac mice grew predominantly *Escherichia coli.* These data suggest that p47^*phox*^ deficiency is associated with increased susceptibility to DSS-induced intestinal inflammation and epithelial permeability.

### p47^phox−/−^ mice have a distinct colonic transcript profile that is not associated with a pattern of leukocyte infiltration during DSS colitis

**Fig. 2:**
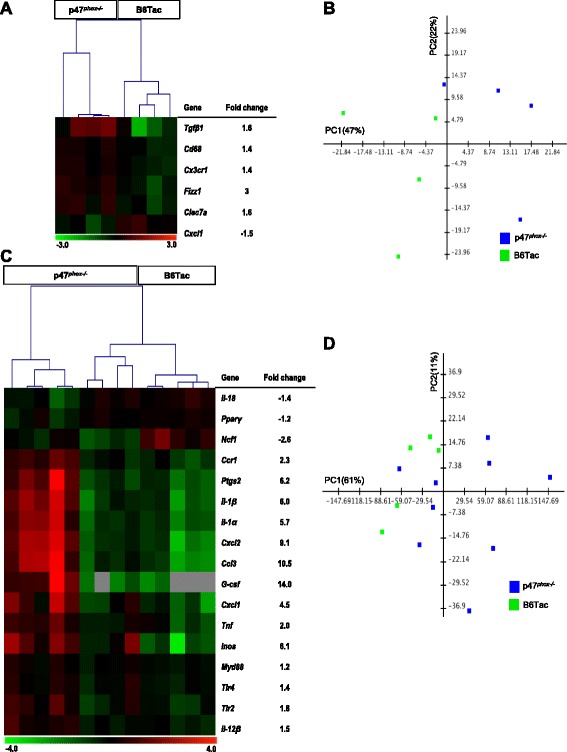
p47^*phox−/−*^ mice have a distinct colonic transcript profile at baseline and during DSS colitis. RNA was isolated from distal colon segments. Normalized transcript counts from nCounter analysis used for heat map reflecting hierarchical clustering of subjects based on differentially regulated transcripts (*p* < 0.05) and the fold change for each transcript comparing p47^*phox−/−*^ versus B6Tac mice at baseline (**a**) and on DSS day 7 (**c**). 2-dimensional PCA of p47^*phox−/−*^ versus B6Tac mice at baseline (**b**) and on DSS day 7 (**d**)

Colons isolated from p47^*phox−/−*^ mice were examined at baseline and after DSS colitis using a custom-designed gene probe panel (Nanostring Technologies). Expression of six genes differed between p47^*phox−/−*^ and B6Tac mice at baseline (Fig. 2a), resulting in independent clustering of p47^*phox−/−*^ mice on principal component analysis (PCA) (Fig. 2b). Similarly, p47^*phox−/−*^ mice with DSS colitis showed a distinct colonic transcript profile and clustered independently of B6Tac mice on PCA (Fig. 2c, d). Colons from p47^*phox−/−*^ mice with DSS colitis were notable for significantly increased expression of granulocyte-colony stimulating factor (*G-csf*), chemokine (C-C motif) ligand 3 (*Ccl3*), and chemokine (C-X-C motif) ligand 2 (*Cxcl2*) compared to controls. Increased transcript levels of the chemokines *Ccl3* and *Cxcl2* may represent polymorphonuclear leukocyte recruitment to the site of inflammation. Thus, to determine whether the increased DSS colitis severity in p47^*phox−/−*^ mice was associated with a distinct pattern of leukocyte infiltration, DSS day 9 colon sections were evaluated using immunohistochemistry (IHC) for the presence of myeloperoxidase (MPO)-producing neutrophils, Mac-1-expressing myeloid cells (monocytes, macrophages, granulocytes), CD3^+^ T cells, and CD138^+^ plasma cells. Although there were slightly more neutrophils, CD3^+^ T cells and plasma cells in the colons from p47^*phox−/−*^ mice with DSS colitis, these differences were not significant (Additional file 3: Figure S3).

### Increased DSS colitis susceptibility in p47^phox−/−^ mice is not reversed by restoration of NOX2-mediated ROS production

**Fig. 3:**
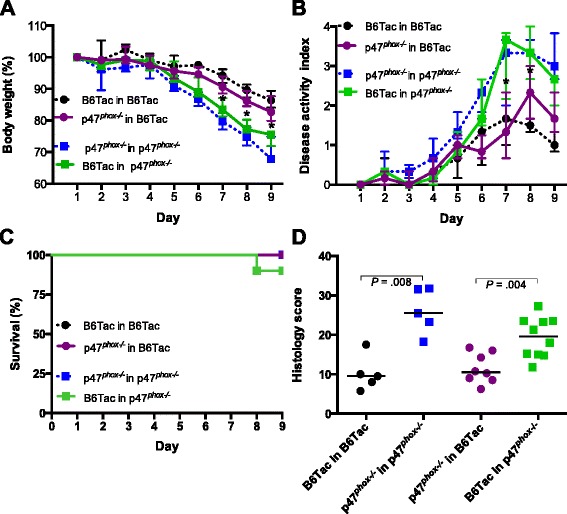
Increased DSS colitis susceptibility in p47^phox−/−^ mice is not reversed by restoration of NOX2-mediated ROS production. BM transplantation was performed in 7-week-old age- and gender- matched irradiated mice (B6Tac-CD45.1^+^ ➔ B6Tac-CD45.1^+^, p47^*phox−/−*^ ➔ p47^*phox−/−*^, B6Tac-CD45.1^+^ ➔ p47^*phox−/−*^, p47^*phox−/−*^ ➔ B6Tac-CD45.1^+^) prior to DSS colitis induction. Changes in body weight (**a**), disease activity index (**b**), and survival (**c**) were assessed (B6Tac-CD45.1^+^ ➔ B6Tac-CD45.1^+^ (*n* = 5); p47^*phox−/−*^ ➔ p47^*phox−/−*^ (*n* = 5); B6Tac-CD45.1^+^ ➔ p47^*phox−/−*^ (*n* = 10); p47^*phox−/−*^ ➔ B6Tac-CD45.1^+^ (*n* = 8); (*p* = 0.615)). **d** Colonic tissue sections were blindly scored for inflammation, depth of injury, and crypt damage on day 9

Given that CGD is characterized by absent phagocyte ROS production, we sought to determine whether restoring NOX2 function would reverse DSS colitis susceptibility in p47^*phox−/−*^ mice. We generated bone marrow chimeras and induced DSS-colitis 10 weeks after successful reconstitution, having achieved >95 % monocyte, neutrophil and B cell donor chimerism, and >80 % T cell donor chimerism in blood (Additional file 4: Figure S4A). Consistent with these findings, we obtained >94 % macrophage and >98 % monocyte donor chimerism in the colonic lamina propria (Additional file 4: Figure S4B). Similar to the phenotype observed in p47^*phox−/−*^ mice and p47^*phox−/−*^ → p47^*phox−/−*^ chimeras, p47^*phox−/−*^ mice reconstituted with B6Tac bone marrow still showed increased susceptibility to DSS colitis compared to B6Tac mice reconstituted with p47^*phox−/−*^ bone marrow. The failure of restoration of p47^*phox*^ activity in the hematopoietic compartment to ameliorate p47^*phox−/−*^ DSS colitis (Fig. 3) suggested that a non-hematopoietic component was critical in mediating the differential susceptibility to DSS colitis.

### p47^phox−/−^ mice have a distinct intestinal microbiome signature before and after DSS colitis

**Fig. 4:**
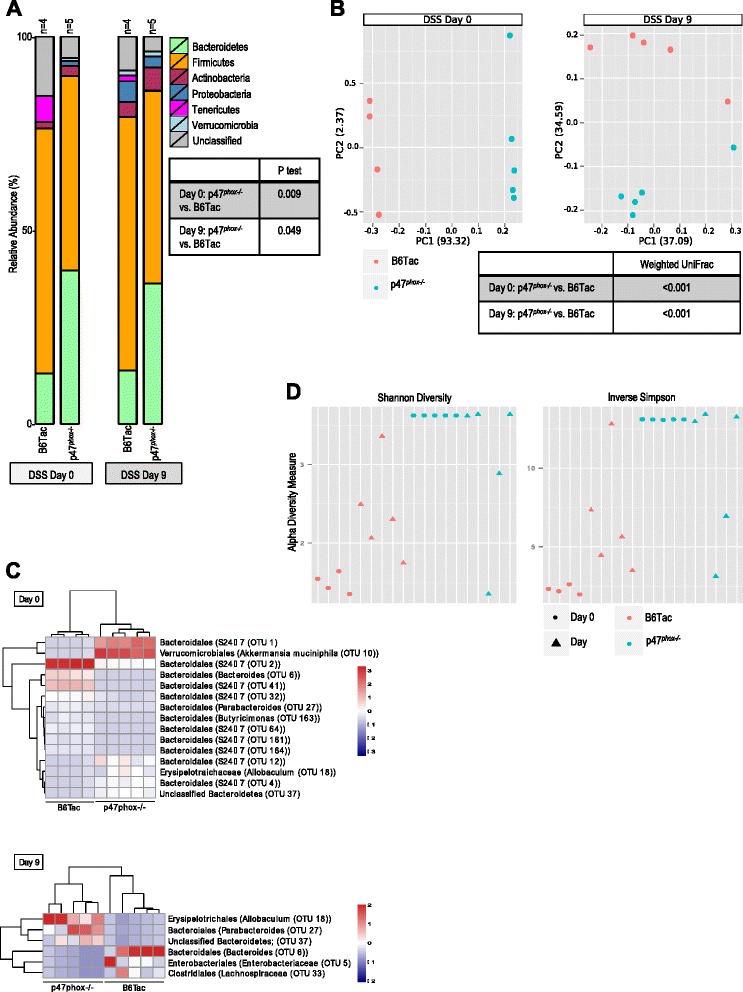
p47^*phox−/−*^ mice have a distinct intestinal microbiome signature before and after DSS colitis. Bacterial taxonomic classifications show colonization with unique taxa and altered representation of diverse taxa in p47^*phox−/−*^ compared to B6Tac mice. **a** Relative abundances of 7 major phyla taxonomies in p47^*phox−/−*^ and B6Tac mice, as well as *p* values from *P* tests are reported for the group comparisons listed for DSS days 0 and 9. **b** PCoA and *p* values of the weighted UniFrac comparing p47^*phox−/−*^ to B6Tac mice on DSS days 0 and 9 are shown. **c** Heat map depicting average relative abundance by LEfSe of bacterial genera in fecal samples from p47^*phox−/−*^ and B6Tac mice on DSS day 0 and day 9. Top quartile of OTUs where comparisons between each experimental group were significant (*p* < 0.05) is shown. Alpha diversity was measured in p47^*phox−/−*^ and B6Tac mice before and after DSS colitis by inverse Simpson and Shannon diversity (**d**)*. Gen*. classified as distinct but unnamed genus in Greengenes reference database, *sp*. designates a distinct species in Greengenes reference database

The involvement of non-hematopoietic elements in susceptibility of p47^*phox−/−*^ mice to DSS colitis, as highlighted by the bone marrow chimera experiments, suggested a possible role for the intestinal microbiome in disease susceptibility. To investigate whether the CGD genotype affects the intestinal microbiome, we analyzed metagenomic 16S rRNA sequences (V1-3 region) of DNA extracted from fecal samples obtained from p47^*phox−/−*^ and B6Tac mice on DSS day 0 (baseline) and DSS day 9. Figure 4a shows the relative abundance of different bacterial phyla in p47^*phox−/−*^ and B6Tac mice on DSS days 0 and 9. For both mouse strains, DSS colitis induction was associated with a relative increase in *Actinobacteria*, *Proteobacteria*, and *Verrucomicrobia* phyla. Fecal microbiome signatures from each experimental group were analyzed by parsimony (*P* test) using the *θ*_YC_ tree generated from operational taxonomic unit (OTU) clusters. We used the weighted UniFrac distance metrics obtained from the phylogenetic tree generated from all samples in the experimental groups to perform principal coordinate analysis (PCoA) (Fig. 4b). p47^*phox−/−*^ and B6Tac fecal microbiome signatures were found to be significantly different (*p* < 0.001) from each other before and after DSS colitis. To determine which taxa were differentially represented between experimental groups, we compared taxonomic abundance using linear discriminant analysis effect size (LEfSe) (Fig. 4c) [6]. The heat maps display the top quartile of bacterial taxa found to have significantly different (*p* < 0.05) relative abundances among the experimental groups examined. *Akkermansia muciniphila* (*Verrucomicrobiales* order) was significantly more abundant in p47^*phox−/−*^ mice at baseline compared to B6Tac mice. On DSS day 9, *Allobaculum* (*Erysipelotrichales* order) and *Parabacteroides* (*Bacteroidales* order) genera were more abundant in p47^*phox−/−*^ mice. The alpha diversity of samples was measured by inverse Simpson and Shannon diversity index (Fig. 4d) and suggested that there was more bacterial richness and diversity in p47^*phox−/−*^ compared to WT mice at baseline. After colitis induction, richness and diversity increased in WT mice but decreased in p47^*phox−/−*^ mice.

### p47^phox−/−^ mice cohoused with B6Tac mice maintain increased susceptibility to DSS colitis

**Fig. 5:**
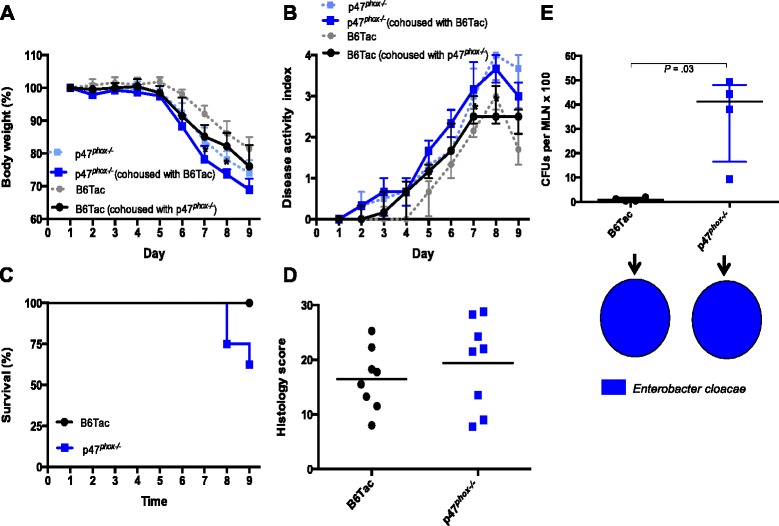
p47^*phox−/−*^ mice cohoused with B6Tac mice maintain increased susceptibility to DSS colitis. Changes in body weight (**a**), disease activity index (**b**), and survival (**c**) were assessed daily (B6Tac cohoused with p47^*phox−/−*^ (*n* = 8); p47^*phox−/−*^ cohoused with B6Tac (*n* = 8), p47^*phox−/−*^ (*n* = 28); B6Tac (*n* = 20)). Significance for comparisons between cohoused mice was determined using the Mann-Whitney *U* test (*p < 0.05) and a log-rank test for survival (*p* = 0.06). **d** Colonic tissue sections were blindly scored for inflammation, depth of injury, and crypt damage on day 9 (B6Tac cohoused with p47^*phox−/−*^ (*n* = 8); p47^*phox−/−*^ cohoused with B6Tac (*n* = 8); (*p* = 0.49)). **e** Bacterial translocation to MLNs was determined on day 9. The number of CFUs per MLN is shown (B6Tac cohoused with p47^*phox−/−*^ (*n* = 4); p47^*phox−/−*^ cohoused with B6Tac (*n* = 4); *p* = 0.03)). *Pie charts* represent the proportion of mice per group where the cultured MLN grew one or more of the listed bacterial species. For all panels, data were generated from 2 independent experiments (except **e**)

**Fig. 6:**
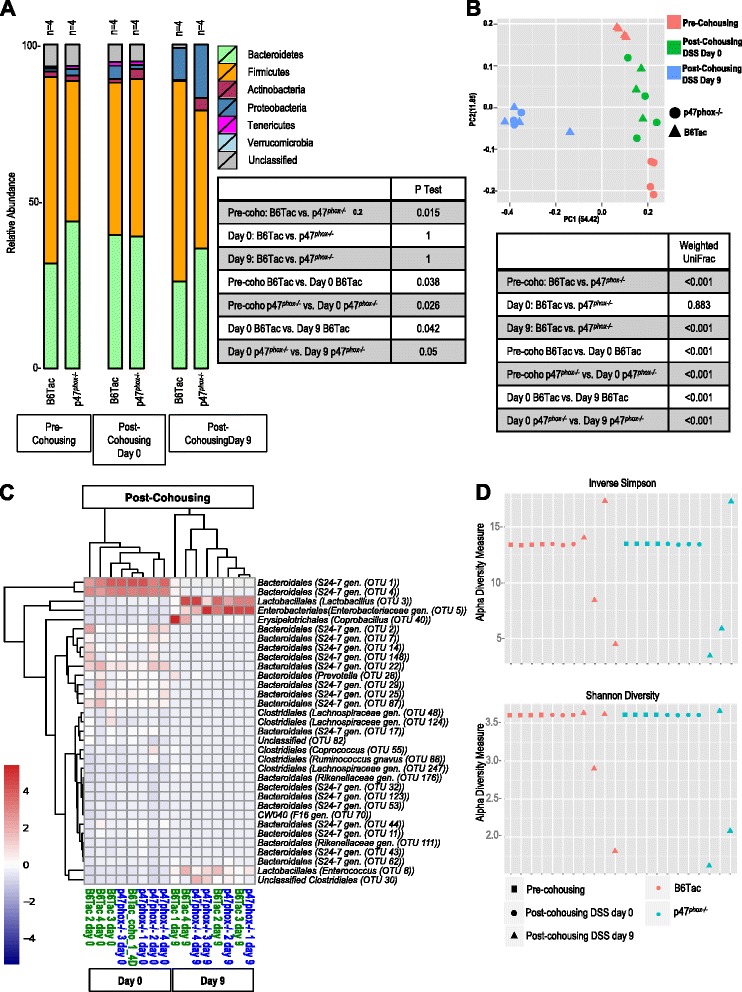
Cohousing homogenizes the intestinal microbiota between p47^*phox−/−*^ and B6Tac mice. **a** Relative abundances of 7 major phyla taxonomies in p47^*phox−/−*^ and B6Tac mice pre-cohousing and post-cohousing (DSS days 0 and 9) are shown. *p* values from *P* tests were used to indicate whether the samples from B6Tac and p47^*phox−/−*^ mice pre-cohousing, on DSS day 0, and DSS day 9, have the same community structure. **b** PCoA and *p* values from weighted UniFrac analyses of p47^*phox−/−*^ and B6Tac mice pre-cohousing and post-cohousing (DSS days 0 and 9) are shown. **c** Heat map depicting average relative abundance by LEfSe of bacterial genera in fecal samples from p47^*phox−/−*^ and B6Tac mice post-cohousing (DSS day 0 and DSS day 9). **d** Alpha diversity was measured in p47^*phox−/−*^ and B6Tac mice pre- and post-cohousing (DSS days 0 and 9) by inverse Simpson and Shannon diversity. *Gen*. classified as distinct but unnamed genus in Greengenes reference database, *sp*. designates a distinct species in Greengenes reference database

To determine whether increased DSS colitis susceptibility in p47^*phox−/−*^ mice was mediated by transferable colitogenic bacteria or could be abated by WT microbiota, we cohoused p47^*phox−/−*^ and B6Tac weanlings for 5 weeks prior to and during the administration of DSS (Fig. 5). In order to confirm that cohousing effectively homogenized the microbiome in the two mouse strains, we performed 16S rRNA sequencing on fecal samples from cohoused p47^*phox−/−*^ and B6Tac mice obtained at three time points: immediately before cohousing (pre-cohousing), after cohousing for 5 weeks (post-cohousing DSS day 0), and after colitis induction (post-cohousing DSS day 9) (Fig. 6). Despite cohousing with B6Tac mice, p47^*phox−/−*^ mice continued to have significantly increased weight loss, colitis severity, mortality, and bacterial translocation during DSS colitis. MLN isolates from both groups only grew *Enterobacter cloacae*. Fecal microbiome sequencing showed that microbiome signatures between B6Tac and p47^*phox−/−*^ mice were significantly different pre-cohousing, but they were not significantly different after cohousing. This finding suggests that cohousing effectively homogenized the microbiome between p47^*phox−/−*^ and B6Tac mice. In Fig. 6c, LEfSe was used to compare taxonomic abundance in cohoused mice before and after DSS colitis. Bacterial richness and diversity as assessed by inverse Simpson and Shannon diversity indices, respectively, were decreased in B6Tac and p47^*phox−/−*^ mice post-DSS colitis compared to before colitis and before cohousing (Fig. 6d). These data suggest that increased DSS colitis susceptibility in p47^*phox−/−*^ mice is not reversed by cohousing with adult WT mice, which may have less-colitogenic intestinal microbiota.

### Homogenizing the intestinal microbiome at birth decreases DSS and *Citrobacter rodentium* colitis susceptibility in p47^phox−/−^ mice

**Fig. 7:**
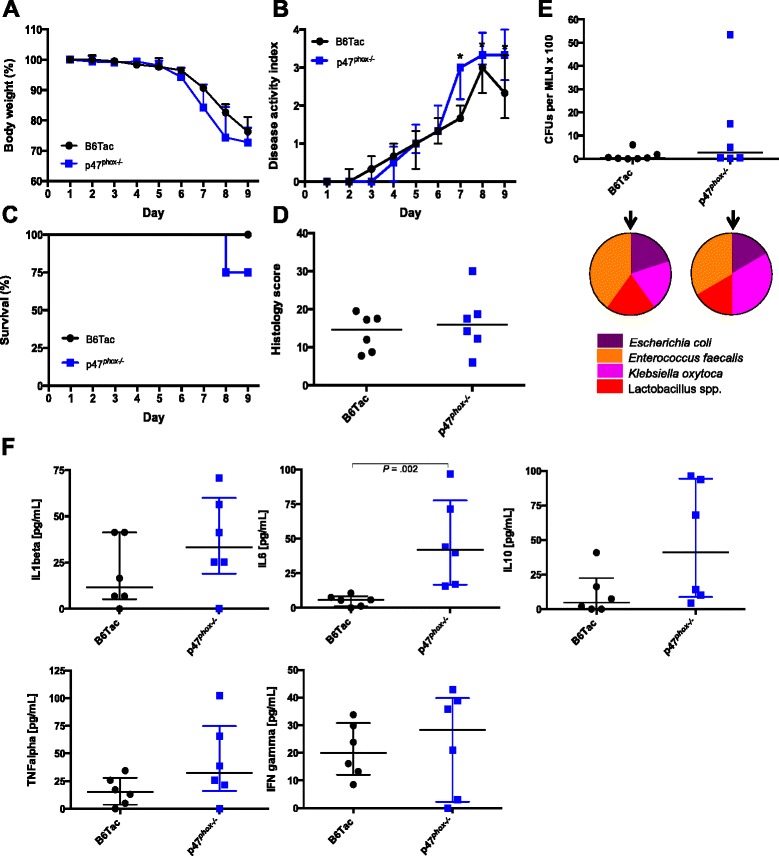
Homogenizing the intestinal microbiome at birth decreases DSS colitis susceptibility in p47^*phox−/−*^ mice. p47^*phox+/−*^ mice were bred, and weanlings were placed in separate cages by gender and genotype (p47^*phox−/−*^ versus B6Tac) prior to DSS colitis induction*.* Changes in body weight (**a**), disease activity index (**b**), and survival (**c**) were assessed daily (B6Tac (*n* = 7); p47^*phox−/−*^ (*n* = 8)). Significance was determined using the Mann-Whitney *U* test (*p < 0.05) and a log-rank test for survival (*p* = 0.17). **d** Colonic tissue sections were blindly scored for inflammation, depth of injury, and crypt damage on day 9 (B6Tac (*n* = 7); p47^*phox−/−*^ (*n* = 6); (*p* = 0.619)). **e** Bacterial translocation to MLNs was determined on day 9. The number of CFUs per MLN is shown (B6Tac (*n* = 7); p47^*phox−/−*^ (*n* = 6)); (*p* = 0.346)). *Pie charts* represent the proportion of mice per group where the cultured MLN grew one or more of the listed bacterial species. For all panels, data were generated from 2 independent experiments (except *E*). **f** Plasma was collected on day 9 for cytokine measurement; *p* values <0.05 are indicated

In order to examine the effects of microbiome homogenization prior to weaning on DSS colitis susceptibility, p47^*phox*+/−^ heterozygous mice were bred, and DSS colitis was induced in p47^*phox−/−*^ mice and littermate controls. With this breeding scheme, DSS colitis susceptibility in p47^*phox−/−*^ mice no longer differed from that in B6Tac littermate controls (Fig. 7). As observed in the cohousing experiments, MLN isolates from both p47^*phox−/−*^ and B6Tac mice grew the same bacteria but with more diversity (*Escherichia coli*, *Enterococcus faecalis*, *Klebsiella oxytoca*, *Lactobacillus* spp.). Although plasma Il1β, Il6, Il10, Tnfα, and Ifnγ levels appeared higher in p47^*phox−/−*^ compared to WT mice, only Il6 levels were significantly elevated. To confirm the phenotype of decreased colitis susceptibility in heterozygously bred p47^*phox−/−*^ mice using an alternate colitis model, we infected littermate p47^*phox−/−*^ and B6Tac mice with *C. rodentium* (Additional file 5: Figure S5). Consistent with our findings using the DSS model, we did not observe increased *C. rodentium* colitis susceptibility in p47^*phox−/−*^ mice compared to littermate B6Tac as evaluated by differences in weight loss, survival, histology, bacterial translocation, and *C. rodentium* fecal load. Of note, while homozygously bred p47^*phox−/−*^ mice show some features of increased susceptibility to *C. rodentium* colitis compared to homozygously bred B6Tac mice, the difference in colitis susceptibility is more modest in this model (Additional file 6: Figure S6).

**Fig. 8:**
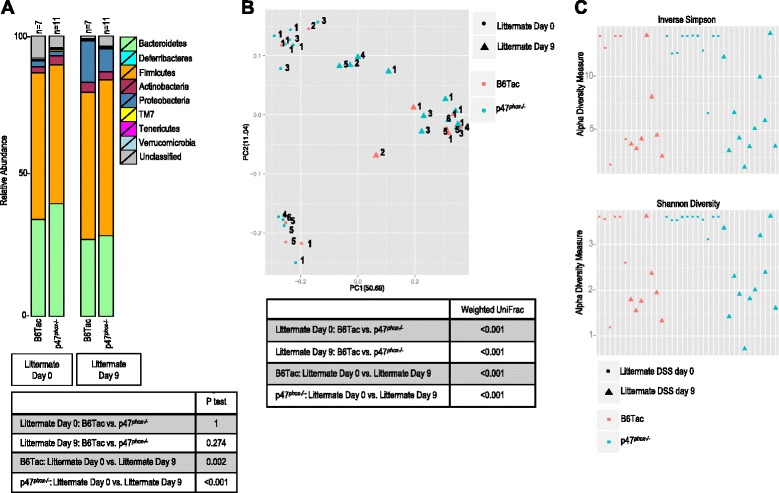
Heterozygous breeding of p47^*phox+/−*^ mice homogenizes the intestinal microbiome in littermates. **a** Relative abundances of 9 major phyla taxonomies in littermate p47^*phox−/−*^ and B6Tac mice on DSS days 0 and 9 are shown. *p* values are reported for the group comparisons listed. **b** PCoA and *p* values from weighted UniFrac analyses of samples from littermate p47^*phox−/−*^ and B6Tac mice on DSS days 0 and 9 are shown. Numbers listed next to each sample reflect the litter to which the mouse generating the sample belonged. **c** Alpha diversity plots measured in littermate p47^*phox−/−*^ and B6Tac mice on DSS days 0 and 9 by inverse Simpson and Shannon diversity are shown

16S rRNA sequencing and subsequent *P* tests using the *θ*_YC_ tree generated from OTU clusters of fecal samples from littermate p47^*phox−/−*^ and B6Tac mice (before and after DSS colitis) showed that the relative abundance of bacterial phyla, and microbiome signatures were similar between littermate p47^*phox−/−*^ and B6Tac mice at baseline and after DSS colitis (Fig. 8a). In contrast, weighted UniFrac analyses suggested that samples from littermate B6Tac and p47^*phox−/−*^ mice were significantly different from each other before and after colitis (Fig. 8b). In particular, p47^*phox−/−*^ and B6Tac microbiome samples on DSS days 0 and 9 clustered into two groups independent of mouse genotype. The weighted UniFrac analysis in these pairings detected a few taxonomic differences, which were likely influenced by litter groupings, thereby emphasizing that it is the microbial signature established from birth to weaning that has the most impact on DSS colitis (and likely *C. rodentium* colitis) susceptibility. LEfSe was used to identify specific taxa with differential abundance in littermate B6Tac versus p47^*phox−/−*^ mice on DSS day 0 and day 9. Before and after colitis, only three and two bacterial taxa, respectively, showed significantly different relative abundance between B6Tac and p47^*phox−/−*^ mice. *Anaerostipes* genus (OTU 99) was significantly more abundant in p47^*phox−/−*^ mice, while *Ruminococcus* genus (OTU 175) and an unclassified S24-7 genus (OTU 83) were less abundant in p47^*phox−/−*^ mice before colitis. Post-colitis, *Streptococcus* genus (OTU 92) was more abundant in B6Tac mice and an unclassified *Lachnospiraceae* genus (OTU 243) was more abundant in p47^*phox−/−*^ mice. Alpha diversity by inverse Simpson and Shannon diversity indices were decreased in samples from most B6Tac and p47^*phox−/−*^ mice post-DSS colitis compared to before colitis (Fig. 8c).

## Discussion

Deficiencies in any of the five subunits of the NOX2 complex cause CGD, and almost half of all CGD patients will develop IBD (unpublished data). Genome-wide association studies have associated the NOX2 subunit genes with IBD and very early-onset IBD [7, 8], suggesting that an understanding of the mechanisms underlying CGD colitis may have broader applications than to this rare immunodeficiency alone. We examined intestinal inflammation in p47^*phox−/−*^ mice using DSS colitis. Surprisingly, restoring phagocyte ROS production did not reduce DSS colitis susceptibility in p47^*phox−/−*^ mice. 16S rRNA fecal sequencing confirmed that homozygously bred p47^*phox−/−*^ and B6Tac mice have distinct microbiome signatures, which can be normalized by cohousing or heterozygous breeding. Cohousing and littermate control experiments uncovered a previously unrecognized contribution of the intestinal microbiome to DSS and *C. rodentium* colitis susceptibility, which is critically established before weaning.

Unlike humans with CGD, p47^*phox−/−*^ mice do not spontaneously develop colitis. We induced colitis using DSS, which denudes the colonic epithelium allowing for bacterial translocation and engaging the innate immune system [9]. We found that the absence of p47^*phox*^ in this setting was not only associated with increased colitis severity but also mortality. Likely causes of death include a combination of dehydration, malnutrition, GI blood loss, bacteremia, and cytokine elevation. Our observed increase in colitis susceptibility in p47^*phox−/−*^ mice is supported by a previous study, which demonstrated increased weight loss, colitis severity, and leukocyte infiltration, as well as localized and systemic cytokine production in p47^*phox−/−*^ mice [10].

The absence of p47^*phox*^ in the mouse colon was associated with a specific gene expression signature before and after the induction of DSS colitis. The gene expression signature post-DSS discriminated p47^*phox−/−*^ from WT mice. It also highlighted the potential roles of chemokines, inflammatory cytokines, and candidate pathways such as PPARγ and IL1α/IL1β. We were limited by the use of a custom-designed gene probe panel, which introduced gene selection bias into the analysis. Since our objective was to detect discriminative signatures, gene expression in whole colons was examined, instead of in specific cell types.

Previous cohousing studies have demonstrated that the intestinal microbiome plays an important role in the development and transfer of DSS colitis susceptibility [11, 12]. It is therefore imperative that the contribution of the intestinal microbiota be teased out from the host genetic predisposition to DSS-induced inflammation. Unsurprisingly, p47^*phox−/−*^ mice had a distinct microbiome signature at baseline, characterized by more abundant *Akkermansia muciniphila*. This gram-negative intestinal mucolytic [13] is reduced in humans with IBD [14, 15]. Moreover, feeding DSS-induced mice *A. muciniphila-*derived extracellular vesicles reduces IBD severity [16]. After DSS colitis induction, *A. muciniphila* was no longer more abundant in p47^*phox−/−*^ mice. Of note, segmented filamentous bacteria, which in mice are correlated with increased secretion of pro-inflammatory IL-17 and decreased regulatory T cells [17], were not differentially abundant in any of the experimental groups studied in each experimental setting. Overall, DSS induction led to somewhat less microbial community structure dissimilarity between mouse genotypes, as well as less bacterial diversity. Cohousing p47^*phox−/−*^ mice with B6Tac mice, and thus exposing p47^*phox−/−*^ mice to WT fecal microbiomes, did not protect p47^*phox−/−*^ mice from developing severe colitis. However, susceptibility to both DSS and *C. rodentium* colitis was significantly reduced when examining heterozygously bred p47^*phox−/−*^ mice and their littermate controls. As expected, 16S rRNA sequencing confirmed that cohousing and heterozygous breeding were effective in normalizing the microbiota between p47^*phox−/−*^ and B6Tac mice. Moreover, in both settings, the strains of translocating bacteria became similar between experimental groups. Nevertheless, it should be noted that 16S rRNA sequencing analysis might not capture discrete differences between bacterial strains that do not vary in abundance between compared experimental groups.

In contrast, restoration of hematopoietic p47^*phox*^ using bone marrow chimeras did not decrease susceptibility to DSS colitis in p47^*phox−/−*^ mice, nor did transplanting p47^*phox*^-deficient cells into WT mice increase their susceptibility to DSS colitis. A potential limitation of these bone marrow chimeras is the radiosensitivity of the components in the organ of interest. We obtained greater than 94 % donor chimerism for hematopoietic cells in the lamina propria. Nevertheless, the colon harbors many stromal cells with immunological functions, as well as stem cells, which are not derived from the hematopoietic compartment [18]. Therefore, future studies will need to examine selective depletion of p47^*phox*^ in specific colonic cell populations. Moreover, although humans with CGD may have improved colitis following hematopoietic stem cell transplantation [19], there may be differences in either the intestinal microbiota or p47^*phox*^-NOX complex interactions in non-hematopoietic colonic cells, which may explain the species-specific differences in colitis remission post-bone marrow transplantation.

## Conclusions

p47^*phox*^-deficient mice have increased DSS-induced intestinal inflammation in association with a unique gene expression signature, which is profoundly affected by the intestinal microbiomic signature established at birth. The CGD genotype shapes the intestinal microbiota, which in turn drives IBD penetrance in this immunodeficiency. Future clinical studies should include characterizing the fecal microbiome in CGD patients at birth in order to elucidate potential candidates for bacteriotherapy.

## Methods

### Mice

p47^*phox*^-deficient mice, B6.129S2-Ncf1^*tm1shl*^N14 (p47^*phox−/−*^), generated as previously described [20], were backcrossed onto the C57BL/6NTac (Taconic Farms) background for 14 generations and inbred in our facility. C57BL/6 NTac (B6Tac) and CD45^+^ congenic B6.SJL (B6Tac-CD45.1^+^) mice were purchased from Taconic Farms and inbred in our facility for ≥2 generations. For littermate experiments, p47^*phox−/−*^ mice were bred with B6Tac mice to generate heterozygous (p47^*phox+/−*^) mice, which were bred together to generate littermate p47^*phox−/−*^ and WT mice. Of note, DSS colitis susceptibility in p47^*phox+/−*^ mice is comparable to that observed in WT mice (data not shown). For cohousing experiments, 4-week-old p47^*phox−/−*^ female weanlings were placed in fresh cages with female age-matched B6Tac mice (1:1 ratio; two mice per cage) for 5 weeks prior to DSS colitis induction while mice remained cohoused. Mice were gender and age (8–11 weeks) matched for each experiment. All mice were housed in aseptic, specific pathogen-free conditions in the same room of the same animal facility at the National Institutes of Allergy and Infectious Diseases (NIAID) (Bethesda, MD), and the NIAID Animal Care and Use Committee approved all experiments. Unless otherwise specified, 3–10 mice were used per experimental group, and all experiments were performed at least twice.

### DSS colitis

Mice were administered filter-sterilized 3.5 % (*w*/*v*) DSS (m.w. 36,000–50,000; MP Biomedicals)-supplemented drinking water ad libitum for 7 days, followed by 1 day of regular autoclaved water. Mice were monitored daily for weight, disease activity, and survival. The DAI was scored as previously described [21]. Briefly, it consists of the sum of the scores attributed to weight loss (0–4), stool consistency (0, 2, 4), and fecal blood (0, 2, 4) divided by 3. Colons (from anus to ilieo-cecal junction) were harvested at indicated time points for histology and RNA extraction.

### *C. rodentium* infection

*C rodentium* suspension was prepared by shaking incubation at 37 °C for 4 h in Luria Broth. Bacterial concentration was assessed by absorbance at an optical density of 600 nm and confirmed by plating of serial dilutions. Mice were inoculated by oral gavage with 5 × 10^9^ colony-forming units (CFU) of *C. rodentium* and monitored for weight and survival. Twelve days post-infection, all mice were euthanized. Sections of spleen and MLNs were analyzed for the presence of viable bacteria. Colons (from anus to ilieo-cecal junction) were harvested on day 12 post-infection for histology. Stool samples were harvested on day 12 and plated on MacConkey agar (Remel) for evaluation of *C. rodentium* fecal load.

### DHR oxidation assay on murine neutrophils

Production of neutrophil-derived ROS was measured using a DHR oxidation assay. Room temperature heparinized blood (300 μL) obtained from B6Tac and p47^*phox−/−*^ mice (one male and one female from each strain) was lysed in filtered flow lysis buffer (NH_4_Cl (0.155 M), KHCO_3_ (0.01 M), EDTA (0.1 mM) in distilled H_2_O) for 5 min and resuspended in flow buffer HBSS with 0.1 % BSA and 0.1 M EDTA. Cells were incubated with DHR and catalase for 5 min and stimulated with PMA (Sigma) for 15 min. Cells were analyzed by flow cytometry using a BD Canto II cytometer (BD Biosciences). Data analysis was performed using FlowJo software (Tree Star).

### Histology and immunohistochemistry

Formalin-fixed colons were paraffin-embedded, and 5-μm-thick sections were either stained with H&E or processed for IHC. H&E-stained colon sections from DSS-treated mice were scored in a blinded fashion as per a modified version of the method described in [22]. Each section was scored for the following parameters: severity of inflammation (0–3), depth of inflammation/injury (0–3), and crypt damage (0–4). Before adding up the scores for each parameter, each score was multiplied by a factor representing the percentage of tissue involved (X 1 for 0–25 %; X 2 for 26–50 %; X 3 for 51–75 %; X 4 for 76–100 %). Thus, the maximum histological severity score in this model is 40. H&E-stained colon sections from *C. rodentium*-infected mice were scored in a blinded fashion as per the method described in [23].

IHC sections were set on poly-l-lysine-coated glass slides, deparaffinized, and rehydrated in graded concentrations of ethanol, followed by heat-induced epitope retrieval. Endogenous peroxidase was blocked using methanol containing 3 % H_2_O_2_ for 15–30 min. Slides were blocked with BSA (Sigma) and incubated overnight at 4° with primary antibody to MPO (Abcam), Mac-1 (Novus biological), CD3 (Abcam), or CD138 (BD Pharmingen). Slides were washed with PBS three times and immunolabeled using the ImmPRESS detection system followed by visualization with ImmPACT Dab peroxidase substrate (Vector). Slides were then counterstained with hematoxylin, mounted with Permount (Fisher), and scanned with a ScanScope (Aperio). Quantification of primary antibody staining was performed by counting the number of stained cells per high-power field (hpf) along the thickness of the same distal colon segment for each mouse.

### Bacterial translocation and identification

MLNs and spleens were aseptically retrieved from mice at baseline and after DSS or *C. rodentium* infection. Tissue was homogenized in sterile HBSS, plated onto sheep blood agar plates (Trypticase Soy Agar with 5 % Sheep Blood, Remel), and incubated at 37 °C for 48 h before CFU quantification.

Microbiological identification of isolates was performed using MALDI-TOF MS (Bruker Daltonics) as previously described [24]. For protein extraction, bacterial colonies in sheep blood agar plate were resuspended in 1 ml 70 % ethanol, vortexed for 1 min, and centrifuged at 13,000 rpm for 2 min. The supernatant was removed completely, and the sample was vortexed for 10 s with 50 μl of 70 % formic acid (FA) and 50 μl acetonitrile (ACN). After 2-min centrifugation at 13,000 rpm, 1 μl of supernatant was spotted onto the target plate and overlaid with 2 μl of alpha-cyano-4-hydroxycinnamic acid (α-CHCA). MALDI-TOF MS analysis was performed using a Microflex LT spectrometer (Bruker Daltonics) and the Biotyper version 4.0.0.1. Manufacturer-recommended cutoff scores of ≥2.0 for species-level identification and ≥1.7 for genus-level identification, and >10 % difference of top score from other genera and species were applied.

### Gene expression

Distal colons were rinsed in RNA*later* (Ambion) and stored at −80 °C in RLT buffer (Qiagen) until RNA isolation. RNA was extracted from homogenized distal colon segments using the RNeasy kit (Qiagen) as per the manufacturer’s protocol. Total RNA (250 ng) was hybridized with reporter and capture probes for a murine custom probe set (Nanostring Technologies) as per manufacturer’s instructions. Samples were prepared on an nCounter Prep station and analyzed on an nCounter Analysis system (Nanostring Technologies). Data were normalized to housekeeping genes and spiked positive controls. Transcript counts less than the mean of the negative control transcripts plus 1 standard deviation (SD) for each sample were considered as background.

### Generation of bone marrow chimeras

Femurs and tibias from donor 7- to 8-week-old p47^*phox−/−*^ (CD45.2^+^) and B6Tac congenic SJL (CD45.1^+^) mice were removed aseptically, and BM was flushed using sterile cold PBS supplemented with 2 mM EDTA. Seven to eight-week-old recipient p47^*phox−/−*^ (CD45.2^+^) and B6Tac-CD45.1^+^ mice were irradiated with 9 Gy and reconstituted 8 h later with 5 × 10^6^ B6Tac-CD45.1^+^ cells (B6Tac-CD45.1^+^ → B6Tac-CD45.1^+^ and B6Tac-CD45.1^+^ → p47^*phox−/−*^ mice) or p47^*phox−/−*^ cells (p47^*phox−/−*^ → p47^*phox−/−*^ and p47^*phox−/−*^ → B6Tac-CD45.1^+^ mice) by lateral tail-vein injection. Mice were given trimethoprim-sulfamethoxazole-supplemented drinking water for the first 4 weeks of reconstitution before being switched to regular drinking water as previously described [25]. Chimeras were treated with DSS 10 weeks after transplantation.

Prior to DSS administration, reconstitution with congenic BM stem cells to a satisfactory level of chimerism was confirmed by assessing the number of CD45.1^+^ (B6Tac-CD45.1^+^) and CD45.2^+^ (p47^*phox−/−*^) leukocytes in the blood and colon using flow cytometry. Colon cell suspensions were generated as described [26]. Cells from blood and colon suspensions were stained with the Live/Dead Fixable Blue staining kit (Invitrogen), followed by Fc blockade with anti-CD16/32 (2.4G2) (eBioscience), followed by staining with antibodies to CD3 (clone 17A2), CD19 (clone 1D3), CD45 (clone 30-F11), CD45.1 (clone A20), CD45.2 (clone 104), MHCII, F4/80 (clone BM8), CD11b (clone M1/70), Ly6C (clone AL-21), Ly6G (clone 1A8) (all antibodies from eBioscience except Ly6C and Ly6G are from BD biosciences). Samples were analyzed on a FACS Fortessa cytometer (BD Biosciences), and data analysis was performed with FlowJo software (Tree Star). In both the blood and the colon, the proportion of donor-derived monocytes, neutrophils, and B cells exceeded 95 %, whereas that of T cells exceeded 80 %.

### Plasma cytokine measurement

Cytokines were measured in mouse plasma using the Bio-Plex® Multiplex Immunoassay based on the Luminex xMAP technology, and data were analyzed using the Bio-Plex® Manager software (Bio-Rad).

### Fecal microbiome analysis

DNA was extracted from sterilely excised stool pellets using PowerSoil® DNA Isolation Kit (MO BIO Laboratories) as per manufacturer’s instructions. The V1-3 region of the 16S rRNA gene was amplified and sequenced using the 454 GS FLX pyrosequencing platform (Roche). The 16S rRNA gene sequences were processed using the mothur software [27] to retain high-quality reads prior to analysis. Preprocessing steps included denoising, trimming, alignment to SILVA 16S rRNA sequence database, pre-clustering, and chimera removal. Sequences were classified using a naive Bayesian classifier trained against a 16S rRNA gene training set provided by the Greengenes Database (May 2013 release). Sequences were then clustered into OTUs using a 3 % distance cutoff with the average neighbor-clustering algorithm. The phyloseq R package [28] was used to plot diversity and richness (Shannon diversity and inverse Simpson calculators, respectively) within samples. To estimate dissimilarity in community structure between pairs of samples, we used the mothur implementation of *θ*_YC_ calculator, which uses the OTU frequency data and subsequently ran parsimony tests (*P* test) to determine significance of differences. UniFrac metrics were calculated using neighbor-joining phylogenetic trees generated using the non-heuristic neighbor-joining algorithm implemented in the software program Clearcut [29] and used as input for the PCoA plots. LEfSe was used to identify features, in this case OTUs, that were statistically different among experimental groups. For each pair of sample groups compared, the non-parametric factorial Kruskal-Wallis sum-rank test detected OTUs with significant differential abundance for each class. A significance alpha of 0.05 and an LDA effect size threshold of 2 were used for all biomarkers reported. A *p* < 0.05 was considered significant. The biomarkers were sorted by LDA score, and those with values in the top quartile were selected to be included in the heat maps. The values used in the heat maps are the relative abundance of the OTUs identified as biomarkers [6]. All graphs were generated using R software (version 3.0.2).

### Statistics

The Mann-Whitney *U* test or, where applicable, a log-rank test (Mantel-Cox) was used to compare values between groups and time points using Prism 6 software (GraphPad Software). A *p* < 0.05 was considered significant. Unless otherwise indicated, numerical values represent medians, and error bars represent inter-quartile range. For gene expression analyses, the differences between experimental groups were assessed by two-tailed Student’s *t* tests with Welch approximation using MultiExperiment Viewer (MeV) software.

### Study approval

All experiments were conducted in accordance with guidelines set forth by the Guide for the Care and Use of Laboratory Animals under a protocol approved by the Animal Care and Use Committee of the NIAID in an Association for Assessment and Accreditation of Laboratory Animal Care-accredited animal facility.

### Availability of supporting data

The raw sequence files supporting the results of this manuscript are available in the NCBI Sequence Read Archive (SRA) under accession number SRP067040.

## Additional files

Additional file 1: Figure S1. p47^*phox−/−*^ mice do not spontaneously develop colitis. H&E staining of representative distal colon segments from 16-month-old p47^*phox−/−*^ mice (*n* = 3) compared to an 8-week-old B6Tac (wild-type) mouse (original magnification ×10). (PPTX 708 kb) Additional file 2: Figure S2. Neutrophils from mice do not produce ROS. DHR oxidation assays performed on PMA-stimulated neutrophils isolated from p47^*phox−/−*^ and B6Tac mice. For each group, female (*n* = 1) and male (*n* = 1) whereas only DHR oxidation assays from female mice are shown. (PPTX 117 kb) Additional file 3: Figure S3. DSS colitis severity in p47^*phox−/−*^ mice is not associated with a pattern of leukocyte infiltration. IHC of MPO^+^, Mac-1^+^, CD3^+^, and CD138^+^ cells was performed on representative distal colon sections from B6Tac and p47^*phox−/−*^ mice (*n* = 4 per group) (original magnification ×10). Scatter dot plots represent the number of stained leukocytes per hpf for each subject. (PPTX 3.09 mb) Additional file 4: Figure S4. Engraftment of BM radiation chimeras before DSS colitis. Whole BM cells from B6Tac (CD45.1^+^) and p47^*phox−/−*^ (CD45.2^+^) congenic mice were cross-transferred to irradiated recipients and evaluated for engraftment 10 weeks later. (*A*) Blood. Each column of FACS plots corresponds to the subpopulation labeled at the top, analyzed for CD45.1 and CD45.2 expression. p47^*phox−/−*^ donor → B6Tac-CD45.1^+^ recipient mice (upper panel); B6Tac-CD45.1^+^ donor → p47^*phox−/−*^ recipient mice (lower panel). Data are representative of 16–20 mice per group. (*B*) Colon lamina propria macrophages. FACS plots of colon lamina propria leukocytes purified from the indicated chimeric mice and gated on MHCIIhiF4/80hiCD11b + cells. Data are representative of 4 mice. (PPTX 379 kb) Additional file 5: Figure S5. Heterozygous breeding decreases susceptibility to *C. rodentium* colitis in p47^*phox−/−*^ mice. Weight (*A*), histology score (colons isolated on day 12 post-infection) (*B*) and survival (*C*) plots of littermate p47*phox−/−* (*n* = 6) and B6Tac (*n* = 3) mice after infection with 5 × 10^9^ CFU of *C. rodentium*. Bacterial load present in MLNs (*D*) and spleen (*E*) was determined on day 12 post-infection. Fecal loads of *C. rodentium* on day 12 post-infection are shown (*F*). Data are representative of two independent experiments. Significance was determined using the Mann-Whitney *U* test (**p* < 0.05) and a log-rank test for survival (*p* = 0.16). (PPTX 199 kb) Additional file 6: Figure S6. Homozygously bred p47^*phox−/−*^ mice have moderately increased susceptibility to *C. rodentium* colitis compared to WT mice. Weight (*A*) and histology score (colons isolated on day 12 post-infection) (*B*) plots of homozygously bred p47^*phox−/−*^ (*n* = 5) and B6Tac (*n* = 5) mice after infection with 5 × 10^9^ CFU of *C. rodentium*. Fecal loads of *C. rodentium* on day 12 post-infection are shown (*C*). Bacterial load present in MLNs (*D*) and spleen (*E*) was determined on day 12 post-infection. Significance was determined using the Mann-Whitney *U* test (**p* < 0.01). (PPTX 170 kb)

## Abbreviations

*Ccl3*: chemokine (C-C motif) ligand 3
CGD: chronic granulomatous disease
*Cxcl2*: chemokine (C-X-C motif) ligand 2.
DSS: dextran sulfate sodium
*G-csf*: granulocyte-colony stimulating factor
H&E: hematoxylin and eosin
hpf: high-power field
IBD: inflammatory bowel disease
LEfSe: linear discriminant analysis effect size
MALDI-TOF MS: matrix-assisted laser desorption ionization-time of flight mass spectrometry
MLN: mesenteric lymph node
MPO: myeloperoxidase
NADPH: nicotinamide adenine dinucleotide phosphate
NIAID: National Institutes of Allergy and Infectious Diseases
NOX2: NADPH oxidase 2
OTU: operational taxonomic unit
*P* test: parsimony test
PCA: principal component analysis
PCoA: principal coordinate analysis
PMA: phorbol 12-myristate 13-acetate
ROS: reactive oxygen species
SD: standard deviation
