# Supplementary material for: Colitis susceptibility in p47phox−/− mice is mediated by the microbiome
Source: Microbiome. 2016 Apr 5;4:13. doi: 10.1186/s40168-016-0159-0 (PMC4820915; doi:10.1186/s40168-016-0159-0)
Supplement: Additional file 2: Figure S2. — Neutrophils from mice do not produce ROS. DHR oxidation assays performed on PMA-stimulated neutrophils isolated from p47phox−/− and B6Tac mice. For each group, female (n = 1) and male (n = 1) whereas only DHR oxidation assays from female mice are shown. (PPTX 117 kb) [file 40168_2016_159_MOESM2_ESM.pptx]

## Slide 1
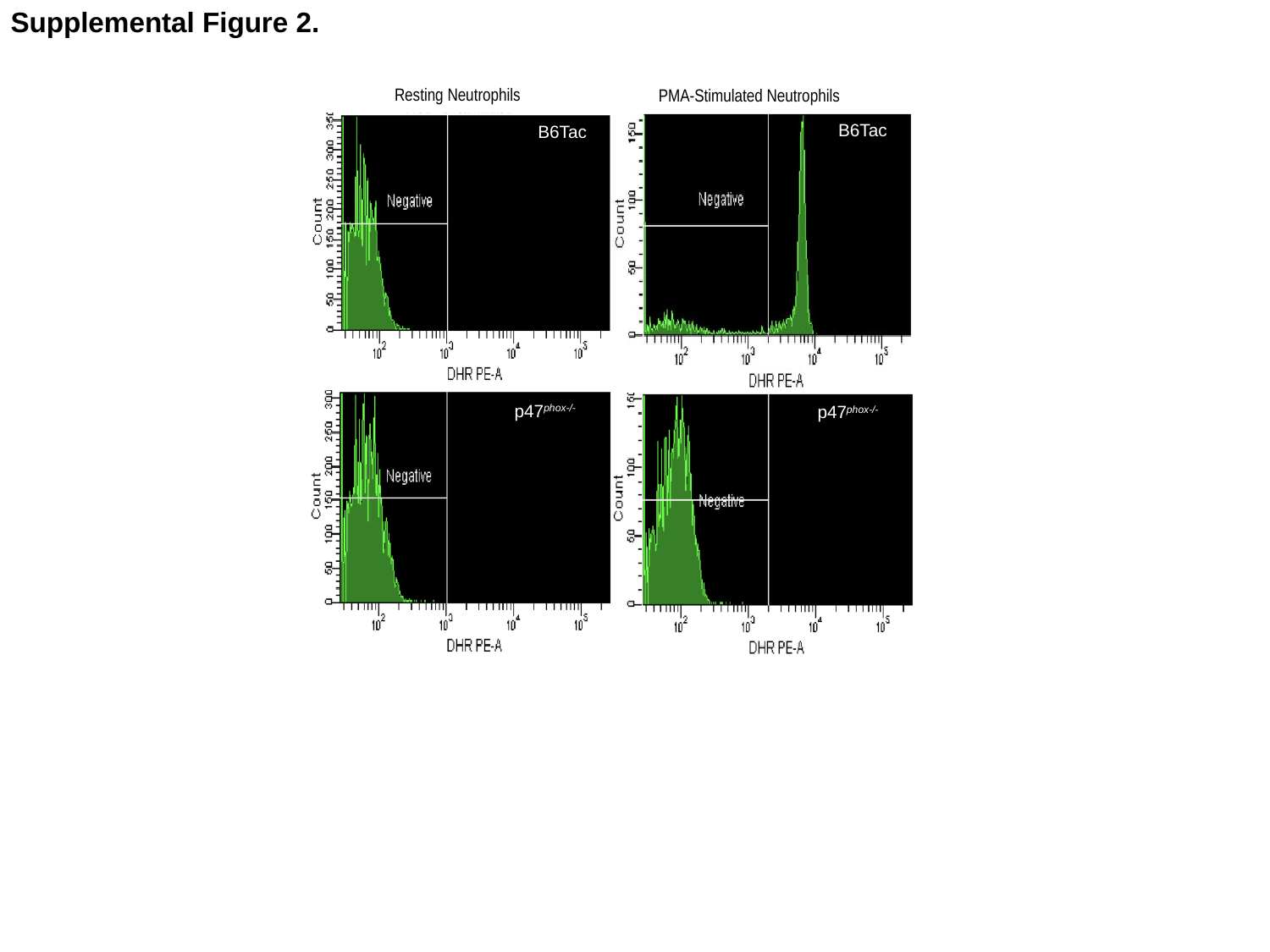

Supplemental Figure 2.
Resting Neutrophils
PMA-Stimulated Neutrophils
p47phox-/-
B6Tac
B6Tac
gp91phox-/-
p47phox-/-
gp91phox-/-
