# Supplementary figures and images for: Colitis susceptibility in p47phox−/− mice is mediated by the microbiome
Source: Microbiome. 2016 Apr 5;4:13. doi: 10.1186/s40168-016-0159-0 (PMC4820915; doi:10.1186/s40168-016-0159-0)

## Slide 1
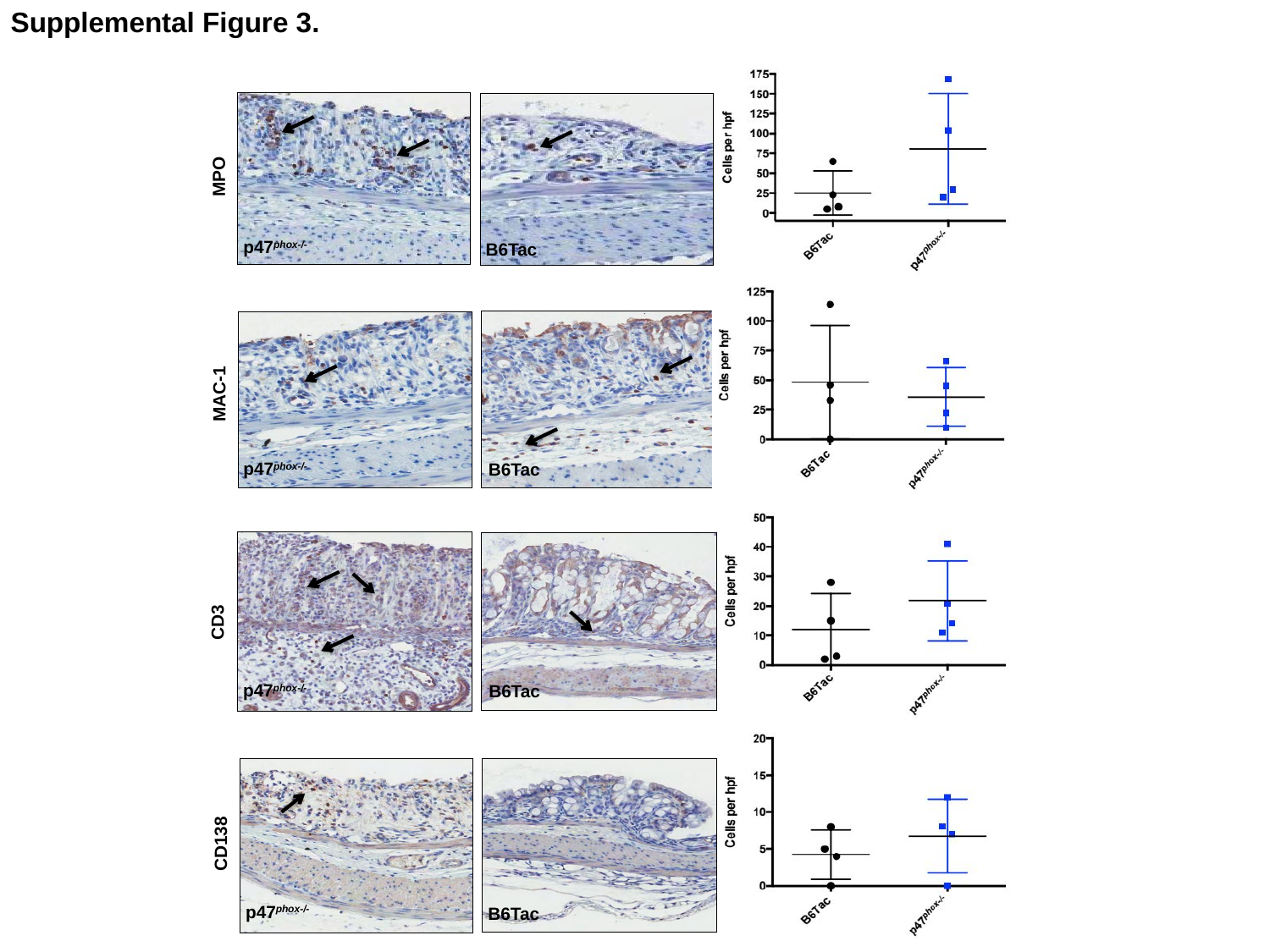

Supplemental Figure 3.
p47phox-/-
B6Tac
MPO
B6Tac
MAC-1
p47phox-/-
B6Tac
p47phox-/-
CD3
B6Tac
p47phox-/-
CD138

Supplement: Additional file 3: Figure S3. — DSS colitis severity in p47phox−/− mice is not associated with a pattern of leukocyte infiltration. IHC of MPO+, Mac-1+, CD3+, and CD138+ cells was performed on representative distal colon sections from B6Tac and p47phox−/− mice (n = 4 per group) (original magnification ×10). Scatter dot plots represent the number of stained leukocytes per hpf for each subject. (PPTX 3.09 mb) [file 40168_2016_159_MOESM3_ESM.pptx]

## Slide 1
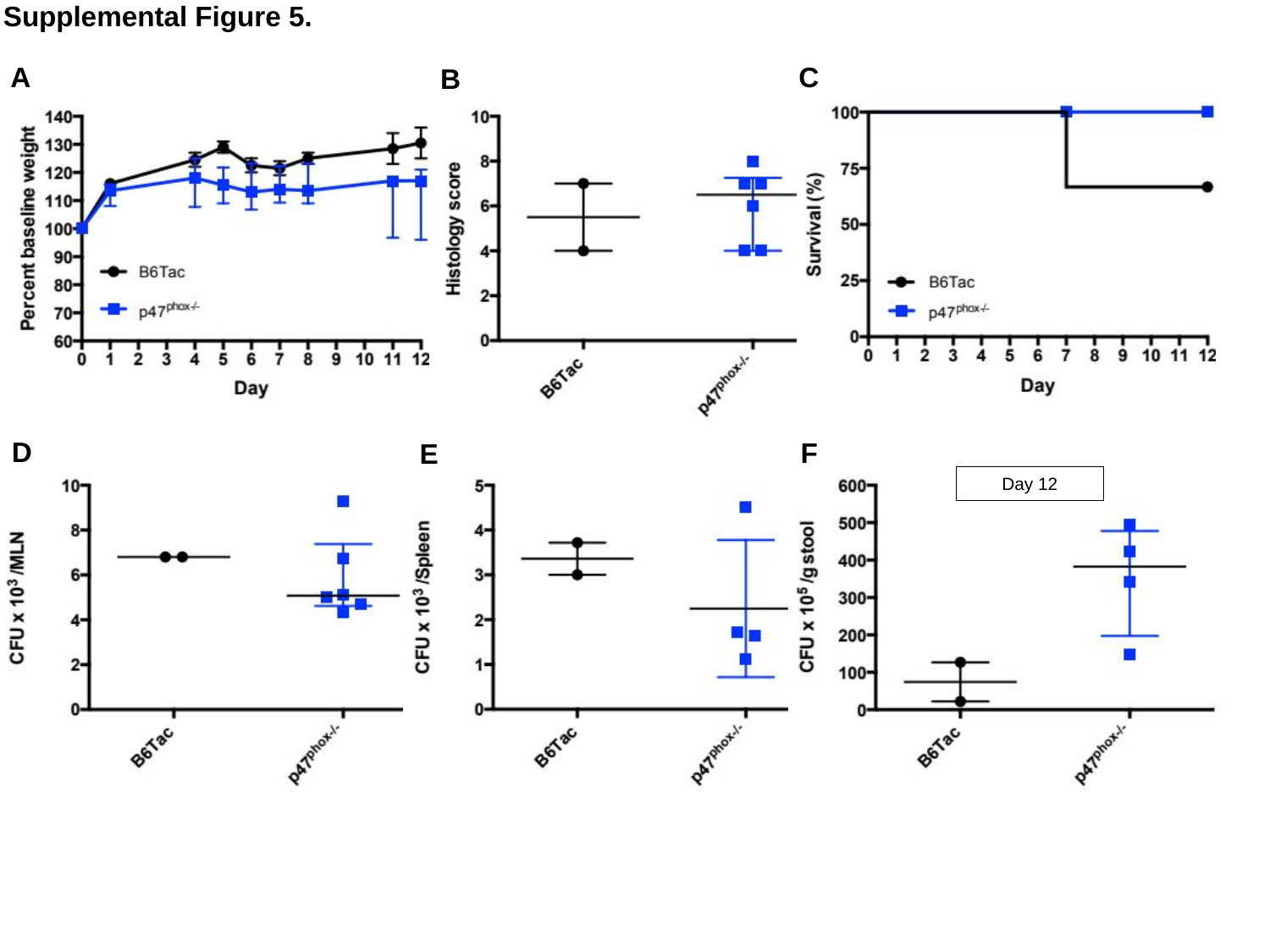

Supplemental Figure 5.
C
A
B
D
F
Day 12
E

Supplement: Additional file 5: Figure S5. — Heterozygous breeding decreases susceptibility to C. rodentium colitis in p47phox−/− mice. Weight (A), histology score (colons isolated on day 12 post-infection) (B) and survival (C) plots of littermate p47phox−/− (n = 6) and B6Tac (n = 3) mice after infection with 5 × 10^9^ CFU of C. rodentium. Bacterial load present in MLNs (D) and spleen (E) was determined on day 12 post-infection. Fecal loads of C. rodentium on day 12 post-infection are shown (F). Data are representative of two independent experiments. Significance was determined using the Mann-Whitney U test (*p < 0.05) and a log-rank test for survival (p = 0.16). (PPTX 199 kb) [file 40168_2016_159_MOESM5_ESM.pptx]

## Slide 1
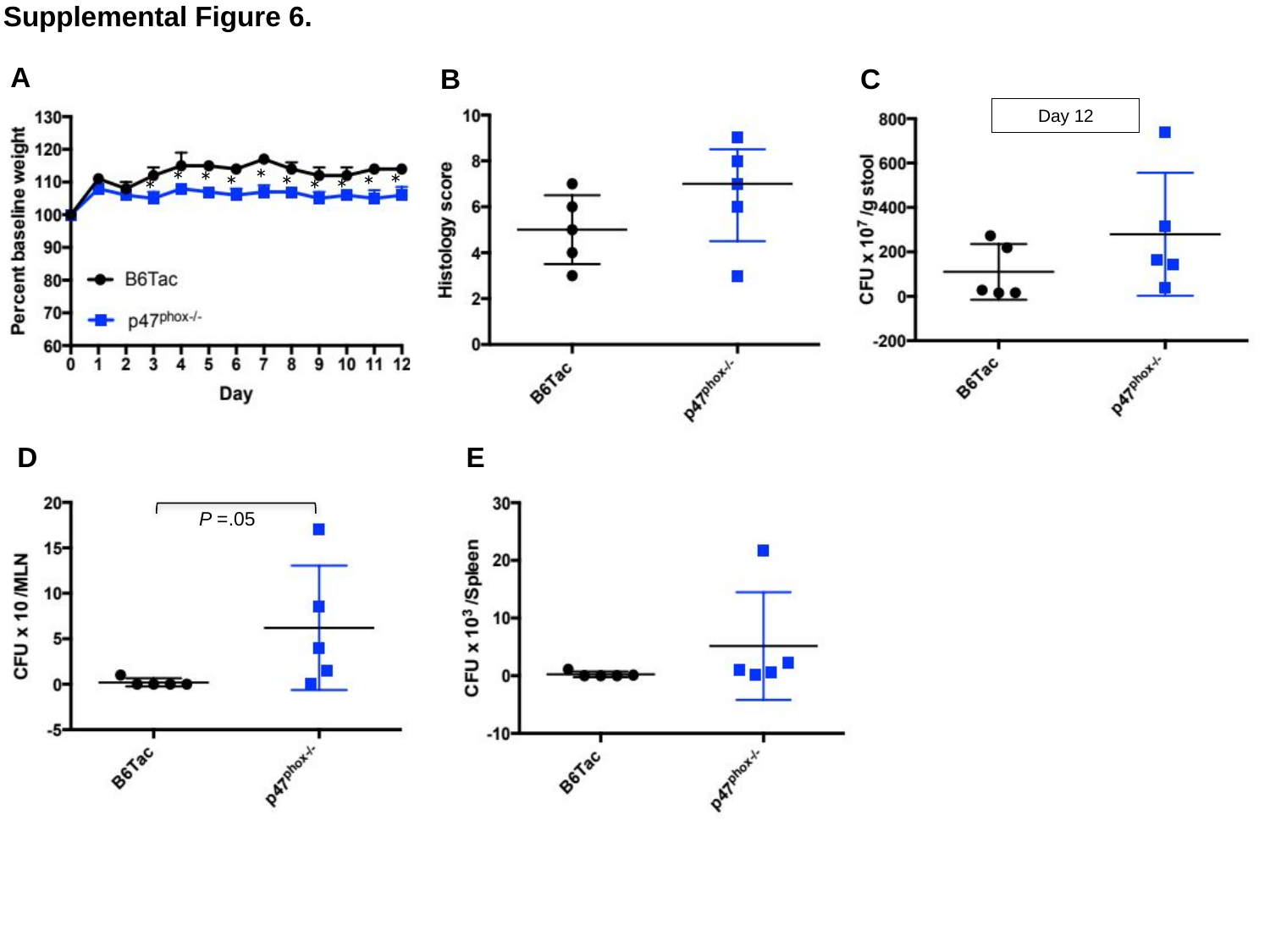

Supplemental Figure 6.
A
B
C
Day 12
*
*
*
*
*
*
*
*
*
*
D
P =.05
E

Supplement: Additional file 6: Figure S6. — Homozygously bred p47phox−/− mice have moderately increased susceptibility to C. rodentium colitis compared to WT mice. Weight (A) and histology score (colons isolated on day 12 post-infection) (B) plots of homozygously bred p47phox−/− (n = 5) and B6Tac (n = 5) mice after infection with 5 × 10^9^ CFU of C. rodentium. Fecal loads of C. rodentium on day 12 post-infection are shown (C). Bacterial load present in MLNs (D) and spleen (E) was determined on day 12 post-infection. Significance was determined using the Mann-Whitney U test (*p < 0.01). (PPTX 170 kb) [file 40168_2016_159_MOESM6_ESM.pptx]
