# Supplementary material for: Colitis susceptibility in p47phox−/− mice is mediated by the microbiome
Source: Microbiome. 2016 Apr 5;4:13. doi: 10.1186/s40168-016-0159-0 (PMC4820915; doi:10.1186/s40168-016-0159-0)
Supplement: Additional file 4: Figure S4. — Engraftment of BM radiation chimeras before DSS colitis. Whole BM cells from B6Tac (CD45.1+) and p47phox−/− (CD45.2+) congenic mice were cross-transferred to irradiated recipients and evaluated for engraftment 10 weeks later. (A) Blood. Each column of FACS plots corresponds to the subpopulation labeled at the top, analyzed for CD45.1 and CD45.2 expression. p47phox−/− donor → B6Tac-CD45.1+ recipient mice (upper panel); B6Tac-CD45.1+ donor → p47phox−/− recipient mice (lower panel). Data are representative of 16–20 mice per group. (B) Colon lamina propria macrophages. FACS plots of colon lamina propria leukocytes purified from the indicated chimeric mice and gated on MHCIIhiF4/80hiCD11b + cells. Data are representative of 4 mice. (PPTX 379 kb) [file 40168_2016_159_MOESM4_ESM.pptx]

## Slide 1
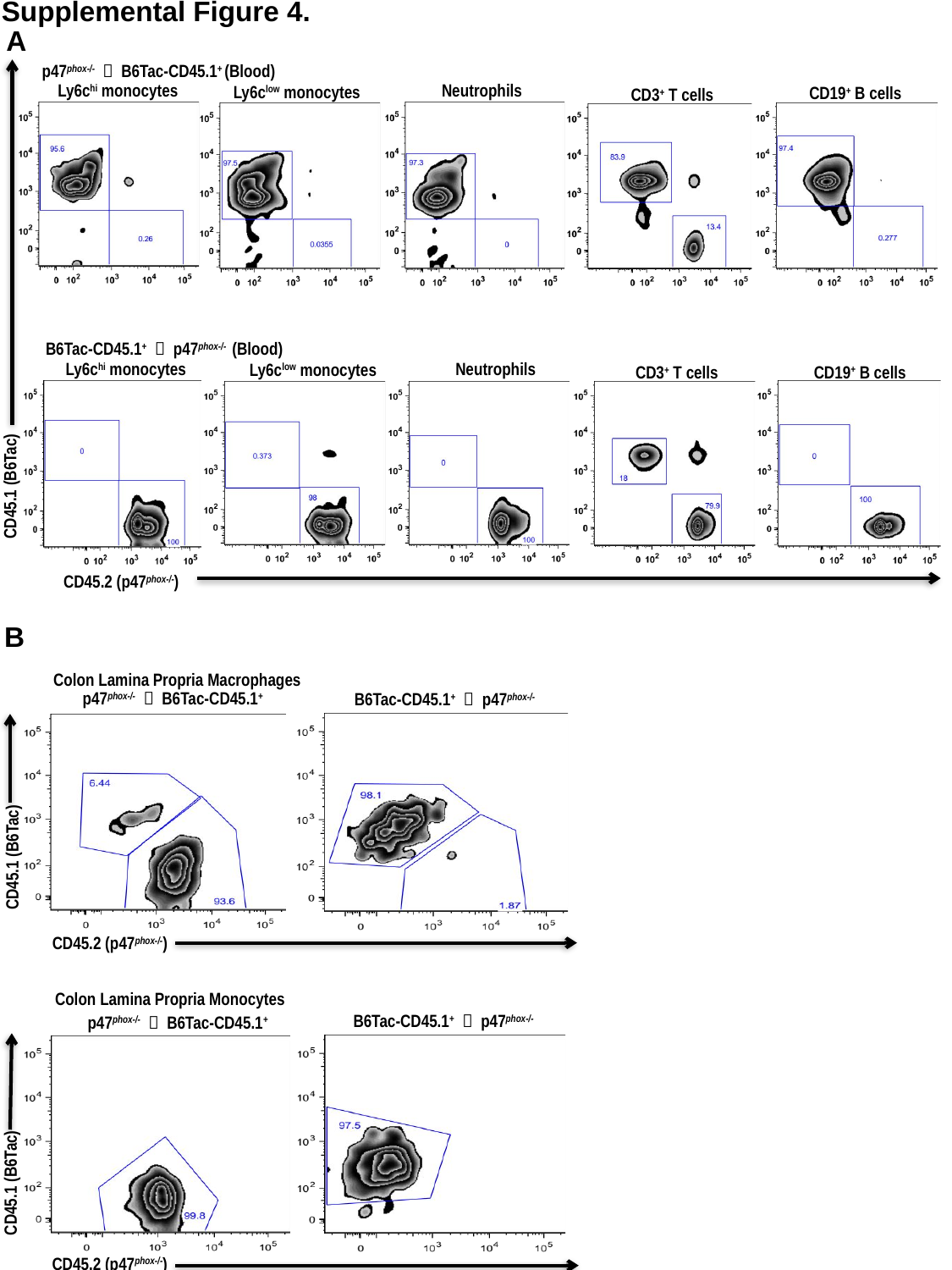

Supplemental Figure 4.
A
p47phox-/-  B6Tac-CD45.1+ (Blood)
CD45.1 (B6Tac)
Neutrophils
Ly6chi monocytes
Ly6clow monocytes
CD19+ B cells
CD3+ T cells
B6Tac-CD45.1+  p47phox-/- (Blood)
Neutrophils
Ly6chi monocytes
Ly6clow monocytes
CD19+ B cells
CD3+ T cells
CD45.2 (p47phox-/-)
B
Colon Lamina Propria Macrophages
p47phox-/-  B6Tac-CD45.1+
B6Tac-CD45.1+  p47phox-/-
CD45.2 (p47phox-/-)
CD45.1 (B6Tac)
Colon Lamina Propria Monocytes
B6Tac-CD45.1+  p47phox-/-
p47phox-/-  B6Tac-CD45.1+
CD45.2 (p47phox-/-)
CD45.1 (B6Tac)
